# Combined *RASSF1A* and *RASSF2A* Promoter Methylation Analysis as Diagnostic Biomarker for Bladder Cancer

**DOI:** 10.1155/2012/701814

**Published:** 2012-03-11

**Authors:** Wei Meng, Alexander Huebner, Ahmad Shabsigh, Arnab Chakravarti, Tim Lautenschlaeger

**Affiliations:** ^1^Department of Radiation Oncology, The Ohio State University, Columbus, OH 43210, USA; ^2^Department of Urology, The Ohio State University, Columbus, OH 43210, USA

## Abstract

Promoter hypermethylation, a widely studied epigenetic event known to influence gene expression levels, has been proposed as a potential biomarker in multiple types of cancer. Clinical diagnostic biomarkers are needed for reliable prediction of bladder cancer recurrence. In this paper, DNA promoter methylation of five C-terminal Ras-association family members (*RASSF1A, RASSF2A, RASSF4, RASSF5*, and *RASSF6*) was studied in 64 formalin-fixed paraffin-embedded (FFPE) bladder cancer and normal adjacent tissues using methylation-specific high-resolution melting (MS-HRM) analysis. Results showed that 73% (30/41) of transitional cell carcinoma, 100% (3/3) of squamous cell carcinoma, and 100% (4/4) of small cell carcinoma demonstrated promoter methylation of the *RASSF1A* or *RASSF2A* gene, but only 6% (1/16) of normal tissues had promoter methylation of *RASSF* genes. Testing positive for hypermethylation of *RASSF1A* or *RASSF2A* promoter provided 77% sensitivity and 94% specificity for identification of cancer tissues with an area under the curve of 0.854, suggesting that promoter methylation analysis of *RASSF1A* and *RASSF2A* genes has potential for use as a recurrence biomarker for bladder cancer patients.

## 1. Introduction

In 2011, about 52,000 men and 17,000 women will be diagnosed with bladder cancer in the United States. Before a normal cell transforms into a bladder cancer cell, a series of molecular alterations are accumulated to initiate the process of transformation. Although we do not fully understand the mechanisms, DNA alterations including hypermethylation and somatic mutation are commonly observed events in human cancer. In a recent bladder cancer study *FGFR3* mutation in combination with *APC*, *RASSF1A*, and *SFRP2* methylation markers provided a sensitivity of 90% using tissue samples and 62% using paired urine samples to identify the presence of cancer with 100% specificity [1]. In nonsmall cell lung cancer (NSCLC) and breast cancer, studies showed that *RASSF1A* had different frequencies of methylation depending on histology [2, 3]. In nasopharyngeal carcinoma, *RASSF2A* was frequently inactivated by its promoter methylation and the methylation correlated with lymph node metastasis [4]. The Ras-association family, also called *RASSF* tumor suppressor genes, currently includes 10 members. All of the *RASSF* proteins contain a Ras-association domain on their C-terminus (*RASSF 1*–*6*) or N-terminus (*RASSF 7*–*10*). Two important issues that are not previously addressed by studies of *RASSF* gene methylation are (1) whether all of the *RASSF* family members show aberrant methylation in bladder cancer and (2) whether methylation pattern of *RASSF* genes can be used as a diagnostic biomarker.

*RASSF1A* (Ras-association domain family 1 isoform A) is the first identified *RASSF* family member which is frequently epigenetically inactivated in a wide range of cancer types. As a tumor suppressor gene, *RASSF1A* regulates the activation of cell death [5], cell cycle [6], and microtubule formation [7]. The methylation signature of *RASSF1A* is thought to be among the earliest cellular changes in tumorigenesis [8]. As a potential tumor suppressor, *RASSF2* plays a role in apoptosis and cell cycle arrest and is frequently downregulated in lung tumor cell lines by hypermethylation [9]. Although the 5′ CpG island of *RASSF3* has been identified earlier, *RASSF3* does not show methylation in glioma tumor cell lines [10]. *RASSF4* is broadly expressed in different human tissues, but its expression is down-regulated by promoter hypermethylation in a majority of tumor cell lines and primary tumors [11]. As a proapoptotic Ras effector, *RASSF5* (*NORE1A*) is frequently inactivated by promoter methylation in human tumors like glioma tumor cell lines, colorectal tumors, and lung cancer [12–15]. *RASSF6* promotes apoptosis by cooperating with activated *K-Ras* to induce cell death and inhibit the tumor cell survival [16]. A high frequency of *RASSF6* methylation is present in leukaemia-related diseases [17]. It appears that all of C-terminal *RASSF* family members have hypermethylation-induced gene inactivation in various types of cancer. While there is extensive literature on *RASSF1A,* other *RASSF* family members have not been studied as widely.

High-resolution melting (HRM) analysis is a new methodology that monitors the melting behavior of PCR amplicons by using DNA intercalating fluorescent dye [18]. Originally the LCGreen was used to develop a closed-tube method for genotyping and mutation scanning [19]. New high sensitive dyes such as EVA Green and SYTO 9 can be used at saturation concentration to monitor the denaturing process of PCR amplicons. Compared to traditional methylation specific PCR (MSP) method, HRM is a reliable and simple method for DNA methylation detection [20, 21].

In this study, to examine diagnostic value of *RASSF* gene methylation, we identified the methylation status of CpG islands associated with C-terminal *RASSF 1*–*6* in a group of formalin fixed paraffin embedded bladder cancer samples using a methylation specific HRM assay.

## 2. Methods

### 2.1. Control and FFPE Tumor Samples

Universal methylated and unmethylated DNA samples (Zymo Research Corp, orange, CA) were used as 100% and 0% methylated control. The methylated DNA was serially diluted in unmethylated DNA to create standard dilutions of 0%, 10%, 50%, and 100% methylated DNA. The standard dilutions from 100% to 0% were used to semiquantitatively measure promoter methylation status of C-terminal *RASSF* genes in FFPE samples.

FFPE blocks from 48 bladder cancer patients were collected by the department of pathology and the Human Tissue Resource Network at The Ohio State University. The study was conducted in accordance with the Institutional Review Board guidelines. We obtained 16 paired tumor and matched normal adjacent tissues and 32 tumor tissues (35 males and 13 females, male-to-female ratio 2.7 : 1; median age 67 years, range 28–90 years). Among these patients, 41 cases were diagnosed with transitional cell carcinoma; 4 cases were small cell carcinoma and 3 cases were squamous cell carcinoma. Clinicopathologic and demographic characteristic of bladder cancer samples are shown in Table 1.

### 2.2. DNA Extraction

DNA samples were extracted using Recover All Total Nucleic Acid Isolation Kit (Life Technologies Corporation, Carlsbad, CA). Briefly, 5–10 mg samples were sliced from paraffin blocks and deparaffinizated by xylene at 50°C, followed by 100% ethanol wash. The air-dry tissue samples were digested by proteinase K for 24 hrs in a microtube shaking incubator set at 50°C. The digested samples were mixed with appropriate volume of isolation additive and 100% ethanol. After passing the mixture through the filter cartridge, the DNA and RNA were retained on the filter. The RNA was removed by on-filter RNase digestion. The DNA was purified by washing buffer and eluted with 95°C nuclease-free water.

### 2.3. Bisulfite Modification

The FFPE DNA bisulfite modification was processed using EZ DNA Methylation Kit (Zymo Research Corp, Orange, CA). The double-stranded DNA was denatured in M-Dilution Buffer for 15 minutes at 37°C, and then CT Conversion Reagent was added to each sample. The samples were incubated in the dark at 50°C for 12 hours followed by 4°C for 10 minutes. After mixing with M-Binding Buffer, the samples were passed through a Zymo-Spin IC Column. The DNA purification and desulphonation were performed on the column. Finally, the bisulfite-modified DNA was eluted by M-Elution Buffer from column matrix.

### 2.4. MS-HRM Primer Design

MS-HRM is based on PCR amplification of bisulfite modified genomic DNA with subsequent HRM analysis of PCR amplicons. The primers were designed to amplify both methylated and unmethylated DNA. Because of DNA degradation in FFPE samples, the sizes of amplicons were limited to 80–180 bp. The free online tool from MethPrimer (http://www.urogene.org/methprimer/index1.html) was used specifically for primer design in this *RASSF* promoter methylation study. Primer sequences and amplicon lengths are shown in Table 2.

### 2.5. High-Resolution Melting Analysis (HRM)

PCR amplification and high-resolution melting analysis were carried out sequentially on a CFX96 real-time PCR system (Biorad, Hercules, CA). PCR was performed in a 20 *μ*L total volume containing: 10 *μ*L 2X Type-it HRM PCR Master Mix (QIAGEN, Hilden, Germany), 1 *μ*L 10 picomol/*μ*L MS-HRM primer, 8 *μ*L nuclease-free water, and 1 *μ*L bisulfite converted DNA (theoretical concentration 10 ng/*μ*L). The amplification consisted of 10 min at 95°C, followed by 40 cycles of 10 s at 95°C, 30 s at annealing temperature and 10 s at 72°C. High-resolution melting analysis were performed at the temperature ramping from 70–95°C by 0.2°C/s and florescence acquisition was set per manufacturer's recommendation.

### 2.6. Statistical Analysis

Methylation was classified as positive if at least 10% methylation was measured. The chi-square test was used to examine the significant differences of methylation depending on histology and staging (*P* ≤ 0.05). To evaluate sensitivity and specificity of methylation as a predictive marker receiver operating characteristic (ROC) analysis was used.

## 3. Results and Discussion

To generate a profile of C-terminal *RASSF* gene epigenetic changes in bladder cancer, 64 bladder FFPE tissue samples were examined by methylation-specific HRM assay. The C-terminal RASSF family members (*RASSF1A*, *RASSF2A*, *RASSF4*, *RASSF5*, and *RASSF6*) were analyzed in this study. In the UCSC Genome Browser, we found a CpG island in the *RASSF3* promoter region. However, due to the dense CG dinucleotides on the CpG island of the identified *RASSF3* promoter, no appropriate HRM primers could be designed for this gene.

### 3.1. Quality Assessment of Methylation-Specific HRM Assay

HRM assay uses double-stranded DNA binding dyes and requires less PCR optimization than other methods. The principle of HRM depends on recording the melting profile of double-stranded DNA samples. As double-stranded DNA is denatured, the fluorescence signal from dye bound to double-stranded DNA decreases. The melting profile is related to amplicon length, DNA sequence and GC content. The high-resolution melting requires smaller temperature increase steps (<0.5°C/s) between each fluorescence reading, which can provide detailed information of amplicon melting behavior.

**Figure 1:**
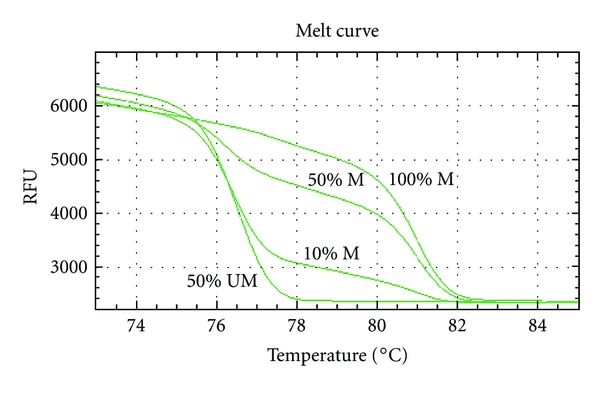
Standard curve constructed for *RASSF* promoter methylation. The dilutions of methylated DNA in unmethylated DNA are as follows: 0% methylation, 10% methylation, 50% methylation, and 100% methylation. Using Meth Primer software, a pair of primers was designed to amplify both methylated and unmethylated sequences after bisulfite conversion. The melting curves of 0% and 100% methylation indicate melting temperature of unmethylated sequence (76.6°C) and methylated sequence (81°C).

MS-HRM is a semi-quantitative method for rapidly assessing the presence of DNA methylation. The standard curve of methylation was used to confirm the *RASSF* gene methylation. The bisulfite-modified fully methylated DNA was diluted in bisulfite-modified fully unmethylated DNA to obtain a series of methylation percentage: 0%, 10%, 50%, and 100% methylation. Only samples containing more than 10% methylation were counted as methylated samples. The standard curve of *RASSF2A* methylation is shown in Figure 1.

### 3.2. *RASSF* Family Member Methylation Profile in Tumor and Normal Adjacent Tissue Samples

**Figure 2:**
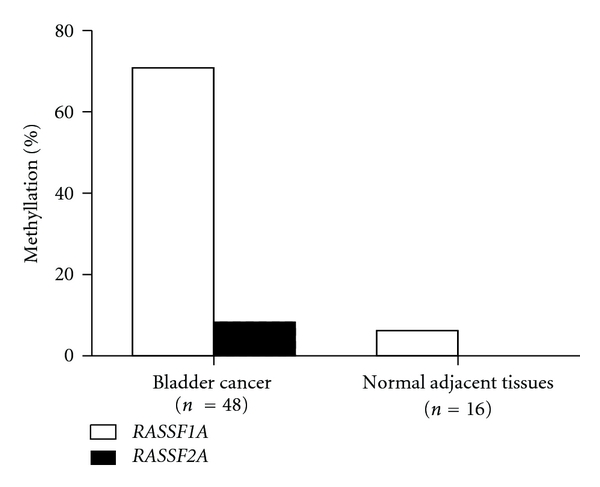
*RASSF1A* and *RASSF2A* methylation profiles of tumor and normal adjacent tissues. 71% (34/48) of tumor samples had *RASSF1A* promoter methylation, and 8% (4/48) had *RASSF2A* promoter methylation. No *RASSF4*, *RASSF5,* and *RASSF6* promoter methylation were detected.

Promoter methylation was analyzed in tumor and normal adjacent tissues from 16 cases using MS-HRM [22]. 56% (9/16) of tumor samples were found to have *RASSF1A* promoter methylation, and 25% (4/16) of tumor samples showed *RASSF2A* promoter methylation, while only 6% (1/16) of the normal adjacent tissue samples showed *RASSF1A* promoter methylation and none of normal adjacent tissue samples showed *RASSF2A* methylation. *RASSF 4*, *5,* and *6* were not found to be methylated in either tumor or normal adjacent tissues.  Figure 2 demonstrates that tumor and normal adjacent tissue showed different *RASSF1A* and *RASSF2A* methylation profiles.

Among the 48 patients with bladder cancer, *RASSF1A* promoter methylation alone had 71% sensitivity and 94% specificity and an area under the curve (AUC) of 0.823 to correctly identify bladder cancer tissue whereas *RASSF1A* and *RASSF2A* together had 77% sensitivity and 94% specificity and AUC of 0.854. *RASSF1A* and *RASSF2A* promoter methylation did discriminate bladder cancer tissue from normal adjacent tissue (*P* < 0.0001). 

### 3.3. *RASSF1A* and *RASSF2A* Methylation Profile in Different Histological Samples

**Figure 3:**
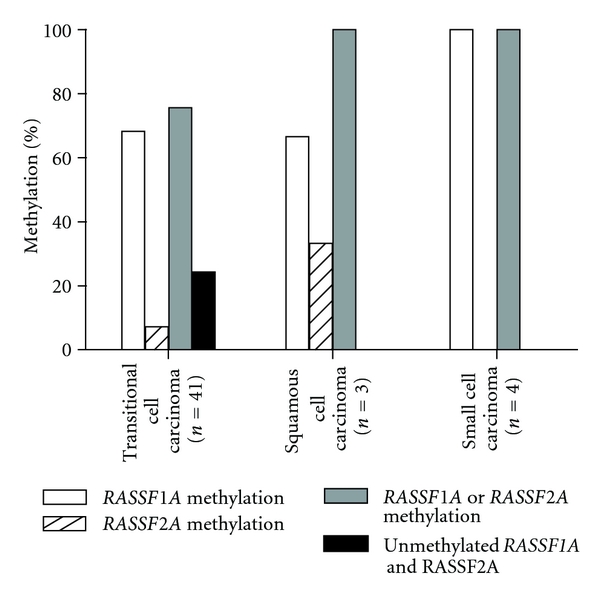
*RASSF1A* and *RASSF2A* methylation profiles of different histology. *RASSF1A* was methylated in 68% (28/41) of transitional cell carcinoma samples, in 100% (4/4) of small cell carcinoma and in 67% (2/3) of squamous cell carcinoma. *RASSF2A* was methylated in 7% (3/41) of transitional cell carcinoma samples, in 0% (0/4) of small cell carcinoma, and in 33% (1/3) of squamous cell carcinoma.

Patient characteristics are summarized in Table 1. 41 patients had tumors with transitional cell carcinoma features 4 with small cell carcinoma, and 3 with squamous cell carcinoma. *RASSF1A* was methylated in 68% (28/41) and *RASSF2A* in 7% (3/41) of transitional cell carcinoma samples. Only one sample had methylation of both the *RASSF1A* and *RASSF2A* promoters. *RASSF1A* was methylated in 100% (4/4) and *RASSF2A* in 0% (0/4) of small cell carcinoma. *RASSF1A* was methylated in 67% (2/3) and *RASSF2A* in 33% (1/3) of squamous cell carcinoma (Figure 3). The frequency of *RASSF1* and *RASSF2* promoter methylation together showed no significant difference with histology in our study (*P* = 0.295).

The reasons that lead to aberrant CpG island methylation of *RASSF1A* and *RASSF2A* in transitional cell carcinoma, small cell carcinoma, and squamous cell carcinoma of the bladder are not well understood. Recent data by Li et al. investigating nonsmall cell lung cancer showed that *RASSF1A* promoter region CpG islands were methylated in 55% of adenocarcinomas, 25% of large cell carcinomas, and 25% of squamous cell carcinomas [3]. This study indicates that promoter methylation of *RASSF* gene family members might be dependent on histology in nonsmall cell lung cancer.

### 3.4. *RASSF1A* and *RASSF2A* Methylation Profile at Different T Stages

**Figure 4:**
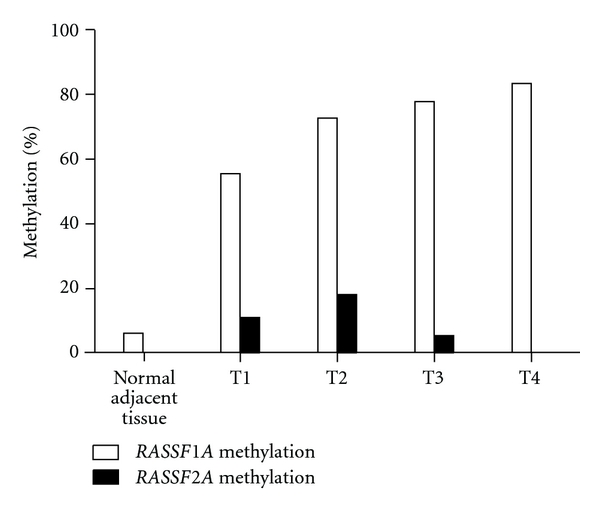
*RASSF1A* and *RASSF2A* methylation profiles in different T stage. *RASSF1A* was only 6% (1/16) in normal adjacent tissues, but percentage of tumor samples with methylated *RASSF1A* genes had a range of 55% −83% from T1 to T4 stage. The *RASSF2A* promoter had a low level (0–18%) at different T stages.

60 malignant bladder tumor and normal adjacent tissue samples from patients with different T stages (16 normal adjacent tissue samples, 9 stage T1, 11 stage T2, 18 stage T3, and 6 stage T4) were analyzed to detect the *RASSF* promoter methylation changes of bladder cancer patients. Methylated *RASSF1A* promoters were only found in 6% (1/16) of normal adjacent tissues. The percentage of promoter methylation positive patients increased with T stage, being lower in T1 tumors and higher with higher stage. The percentage of samples with methylated *RASSF1A* genes was 55% (5/9) in stage T1, 73% (8/11) in stage T2, 78% (14/18) in stage T3, and 83% (5/6) in specimens from T4 tumors (Figure 4). The frequency of *RASSF1* and *RASSF2* promoter methylation was not associated with T stage (*P* = 0.363). Due to lack of samples with *RASSF2A* methylation, no association between *RASSF2A* methylation and T stage was identified in the current data set. Similar results were found in a recent lung cancer study [23]. The *RASSF2A* promoter methylation was found at low levels (0–18%) at different T stages.

### 3.5. *RASSF1A* and *RASSF2A* Methylation Profile at Different N Stages

**Figure 5:**
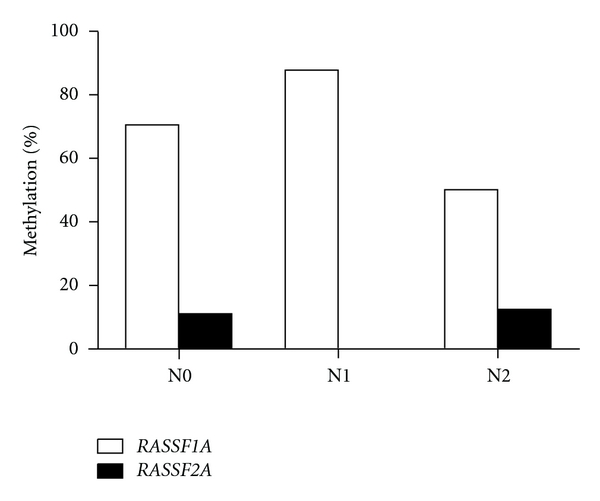
*RASSF1A* and *RASSF2A* methylation profiles in different N stages. Percentage of *RASSF1A* methylation had a range of 50–87.5% in different N stage, and percentage of *RASSF2A* had range of 0% −11%. Aberrant methylation of *RASSF1A* and *RASSF2A* promotor showed no relationship to lymph node metastasis.

We examined methylation status of *RASSF1A* and *RASSF2A* in 27 bladder tumor samples with stage N0, 8 samples with stage N1, and 8 samples with stage N2. Percentage of *RASSF1A* promoter methylation had a range of 50–87.5% in tissues from patients with different lymph node metastasis stage. The percentage of samples with methylated *RASSF2A* was 11% (3/27) for stage N0, and 6% (1/16) for stage N1/2 (Figure 5). Based on the result of chi-square test, there is no significant difference between the frequency of *RASSF1* and *RASSF2* promoter methylation in lymph node positive and negative patients.

In nasopharyngeal carcinoma, aberrant methylation of *RASSF2A* promotor was found to be associated with lymph node metastasis [24]. Although both *RASSF1A* and *RASSF2* protein can function as a negative effector of Ras protein in tumor formation, *RASSF2A* and *RASSF1A* have apparently different functions in different type of tumors.

### 3.6. *RASSF4*, *RASSF5*, and *RASSF5* Methylation

*RASSF1-6* share a variable N-terminal sequence followed by a Ras-association domain [25]. The HRM analysis showed no detectable promoter methylation of *RASSF4*, *RASSF5* and *RASSF6* in bladder cancer and normal adjacent tissue samples.

## 4. Discussion

DNA methylation and histone modification are widely studied epigenetic events. Promoter hypermethylation has been proposed as a potential diagnostic or prognostic biomarker in various cancers. Recent research showed that urine is potentially useful for bladder cancer screening [26, 27]. Methylation status of certain genes identified in urine samples showed higher sensitivity than the conventional urine cytology method. These studies indicated that detection of promoter methylation in urine specimen could potentially provide a simple, noninvasive, and sufficiently sensitive method for bladder cancer screening in the future.

In our study, a new methodology, methylation-specific-high resolution melting analysis was used to examine the melting behavior of methylated or unmethylated *RASSF* gene amplicons. This provides a simple and reproducible method for promoter methylation assessment. We studied DNA promoter methylation of five *RASSF* family members (*RASSF1A*, *RASSF2A*, *RASSF4*, *RASSF5,* and *RASSF6*) in FFPE bladder cancer tissues and normal adjacent tissues. We identified distinctive *RASSF1A* and *RASSF2A* gene promoter methylation profiles that differentiate between bladder cancer and normal adjacent tissue samples. Using *RASSF1A* and *RASSF2A* genes together showed an acceptable sensitivity (77%) and high specificity (94%) identifying bladder cancer tissues. Previous studies have identified *RASSF1A *promoter methylation as a potentially useful urine biomarker for the presence of invasive bladder cancer [26, 28, 29]. We now show that the addition of *RASSF2A *promoter methylation analysis can improve the sensitivity potentially without compromising specificity. There was no significant correlation of *RASSF1A *methylation with histology and N stage. As discussed by Serizawa [1], results also showed that *FGFR3 *mutation in bladder cancer when combined with methylation markers (*APC*, *RASSF1A *and *SFRP2*) provided a sensitivity of 90% to identify bladder tumors. It remains to be shown if the addition of an *RASSF2A *promoter methylation assay to the previously published urine biomarker assay can indeed improve sensitivity when using urine samples. Limitations of the study include small sample size or lack of a validation data and sample set. In addition we did not have sufficient data to analyze if *RASSF* gene family promoter methylation could predict the recurrence of bladder cancer.

Methylation analysis of both *RASSF1A *and *RASSF2A *genes appeared to increase the sensitivity of discriminating cancer from normal adjacent tissue. The addition of *RASSF2A *methylation analysis to recent bladder cancer biomarker signatures has the potential to further increase sensitivity for bladder cancer diagnosis. *RASSF1A *and *RASSF2A *promoter methylation analysis could be useful as a biomarker to detect the presence of bladder cancer recurrence.

## Figures and Tables

**Table 1:** Patient demographic information.

	Study populations		
	Total (*N* = 64)	Bladder cancer tissue specimen (*n* = 48)	Matched bladder cancer and normal adjacent tissues (*n* = 16)
Age (years): mean (range)	68 (28–90)	67 (28–90)	69 (53–90)

Gender			
Male	35	35	12
Female	13	13	4

Histologic cell type			
Transitional cell carcinoma	41	41	12
Small cell carcinoma	4	4	1
Squamous cell carcinoma	3	3	3

T stage			
T1	9	9	2
T2	11	11	3
T3	18	18	8
T4	6	6	1
Tx	4	4	4

N stage			
N0	27	27	9
N1	8	8	2
N2	8	8	3
Nx	5	5	2

**Table 2:** Primer sequences and melting temperature for *RASSF* genes.

Analysis	Sense primer	Antisense primer	Product size (bp)	*T*_anneal_ (°C)	Unmethylation *T*_melt_ (°C)	Methylation *T*_melt_ (°C)
*RASSF1A*	ATGTTAAGGGAATTTATTTAGAATGTATTT	AACCTTCACTTAAAATAAAAAAAA	146	53	73.2	75.6
*RASSF2A*	GAAGGGGTAGTTAAGGGGTAG	CCTCTACTCATCTATAACCCAAATAC	176	55	76.6	81
*RASSF4*	TAAATGGTTTGTGGTTTTTTGTTTT	AAAAACACCTTTATACAATCTAACC	175	55	72.8	74
*RASSF5*	GAAAGAGGTAGGGTTGAAGGTTTAG	TCACCTAAAACAACTACAAAATTCC	105	55	74	75.6
*RASSF6*	GTTTAGTTGAGTTATGTTTTGGGAG	AAAAAACCAATACCCTATCTCTACC	126	55	75.8	79.2

*T*_anneal_, primer annealing temperature

*T*_melt_, amplicon melting temperature.
